# Enhanced Tolerance against a Fungal Pathogen and Insect Resistance in Transgenic Tobacco Plants Overexpressing an Endochitinase Gene from *Serratia marcescens*

**DOI:** 10.3390/ijms20143482

**Published:** 2019-07-16

**Authors:** Samantha Sarai Navarro-González, José Augusto Ramírez-Trujillo, Guadalupe Peña-Chora, Paul Gaytán, Abigail Roldán-Salgado, Gerardo Corzo, Laura Patricia Lina-García, Víctor Manuel Hernández-Velázquez, Ramón Suárez-Rodríguez

**Affiliations:** 1 Centro de Investigación en Biotecnología, Universidad Autónoma del Estado de Morelos, Av. Universidad 1001, Col. Chamilpa, Cuernavaca, Morelos 62209, Mexico; 2 Centro de Investigaciones Biológicas, Universidad Autónoma del Estado de Morelos, Av. Universidad 1001, Col. Chamilpa, Cuernavaca, Morelos 62209, Mexico; 3 Instituto de Biotecnología, Universidad Nacional Autónoma de México, Av. Universidad 2001, Col. Chamilpa, Cuernavaca, Morelos 62210, Mexico

**Keywords:** *Serratia marcescens*, bacterial chitinase, antifungal activity, transgenic plants, *Spodoptera frugiperda*

## Abstract

In this study we cloned a chitinase gene (*SmchiC*), from *Serratia marcescens* isolated from the corpse of a *Diatraea magnifactella* lepidopteran, which is an important sugarcane pest. The chitinase gene *SmchiC* amplified from the *S. marcescens* genome was cloned into the transformation vector p2X35SChiC and used to transform tobacco (*Nicotiana tabacum* L. cv Petit Havana SR1). The resistance of these transgenic plants to the necrotrophic fungus *Botrytis cinerea* and to the pest *Spodoptera frugiperda* was evaluated: both the activity of chitinase as well as the resistance against *B. cinerea* and *S. frugiperda* was significantly higher in transgenic plants compared to the wild-type.

## 1. Introduction

Productivity of crops grown for human consumption is at risk due to the incidence of pathogens and pests. Crop losses due to these harmful organisms can be substantial, and they may be prevented, or reduced, by crop protection measures [1]. Food supply for the population of billions of people depends on the effective protection of crops and animals from pests. The chemical control of pests was efficacious and attractive during the forties and fifties of the last century. However, the negative effects of the use of chemicals quickly became evident because of their adverse effects and the accumulation in soil, water, air, agricultural products and in the body fat of animals. In addition, the development of resistance in target organisms makes it necessary to search for more selective and environmentally acceptable agents for pest control [2,3]. Due to the importance of the chitinolytic enzymes in the growth and development of insects, nematodes and fungi, they are of note with respect to their development as biopesticides or defense proteins in transgenic plants and pest control agents. In this context, glycosyl hydrolase enzymes have the ability to hydrolyze the unbranched polymer of chitin comprised of β-1,4-N-acetylglucosamine (GlcNAc), the second most abundant polymer in nature, after cellulose [4], GlcNAc is widely distributed in nature in the outer skeleton of insects and in internal structures, as well as in fungi, yeasts, algae, crabs, shrimps, and plants [5].

Plant chitinases are classified as pathogenesis-related proteins (PR) as they are induced after biotic or abiotic stresses [6,7]. However, one of the most widely biotechnology strategies used for biological control is to overexpress genes such chitinases and glucanases from various sources in transgenic plants [8,9], so it is important to study its applicability in the improvement of plant health [10,11] as an alternative to the use of chemicals. Such enzymes are thought to play a dual role, both by inhibiting fungal growth by cell wall digestion and by releasing pathogen-borne elicitors that induce further defense reactions in the host [7,12]. Transgenic plants overexpressing chitinases from different sources have demonstrated enhanced levels of resistance to fungal infection, and also have delayed disease symptoms when challenged with fungal pathogens [13,14,15,16].

One of the first studies using hydrolytic enzymes was a bean chitinase, which inhibited the growth of the fungal pathogen *Trichoderma viride* [17]. Furthermore, transgenic tobacco seedlings constitutively expressing a bean chitinase gene under control of the cauliflower mosaic virus 35S promoter showed that seedlings had an increased ability to survive in soil infested with the fungal pathogen *Rhizoctonia solani.* Here, susceptibility to infection ranged from 22.7 to 37.1% in transformed plants in comparison to untransformed plants with 53% mortality [18]. Tobacco plants overexpressing a chitinase gene *EuCHIT2* from *Eucommia ulmoides* showed circumstantial evidence that this hydrolytic enzyme increased the resistance against *Erysiphe cichoracearum* DC [19]. 

In addition to fungal control, to minimize the adverse effects of pesticides, different subspecies of *Bacillus thuringiensis* are the most well-known microbial entomopathogens that produce insecticidal proteins which specifically targets insect pests, particularly lepidoptera [20,21]. Although these strategies have reduced adverse effects compared to chemical insecticides, the development of resistance to the insecticidal proteins has raised concerns [22]. 

Chitinolytic bacteria have been correlated with insect biocontrol [23] and considered as a source of useful bioactive molecules and genes for this application [24]. Insect chitinases play a critical role in insect growth and development, and their overexpression at appropriate times could disrupt the cuticle and/or gut physiology in many insect species leading to negative effects and even death [25]. In fact, the usage of Gram negative bacterial strains like *S. marcescens* has been observed to be a great biocontrol agent against *Spodoptera litura* at different developmental stages [24,26], due to its ability to produce different chitinolytic enzymes [27]. In this way, the search for chitinolytic enzymes produced in transgenic plants with insecticidal activity is of scientific interest. For example, the expression of an insect chitinase from the cotton leaf worm, *S. littoralis*, in maize plants improved its tolerance against insects [28]. Studies of chitinase A (ChiA) protein from *Autographa californica* multicapsid nucleopolyhedrovirus (AcMNPV) demonstrated the ultimate liquefaction of infected host larvae [29]. 

In the present work we developed plants with stress tolerance by evaluation of transgenic tobacco lines harboring endochitinase *chiC* gene from *S. marcescens* (*SmChiC*) for resistance to the gray mold *Botrytis cinerea* (*B. cinerea)* and to the insect pest *S. frugiperda*. Three transgenic tobacco lines expressing constitutively endochitinase were assayed for infection of plants, antifungal inhibition on crude protein extracts, and by insect feeding toxicity assays. The results included in this study demonstrate that expression of this endochitinase gene enhanced tolerance to both the fungus and insect, compared with non-transformed plants. 

## 2. Results

### 2.1. Bacteria Strain Identification and Phylogenetic Analysis

**Figure 1:**
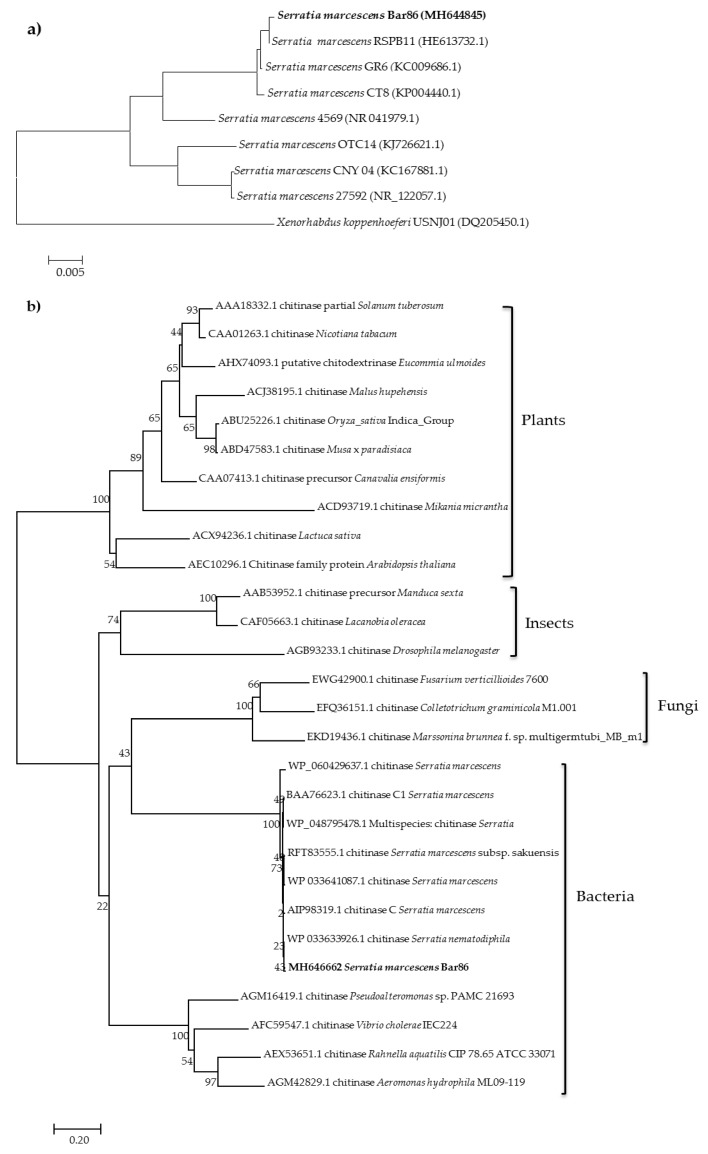
Phylogenetic analysis. (**a**) Relationships showing the relatedness of 16S rDNA between the new isolate of *Serratia marcescens* Bar86 and selected reference isolates derived from the GenBank database. (**b**) Phylogenetic tree of deduced SmChiC amino acid sequences with chitinases from plants, insects, fungi and bacteria.

Identification of the strain isolate Bar86 was based on its 16S rDNA gene sequence, and it showing 99% identity with other *S. marcescens* in the existing database (Figure 1a). Figure 1b shows the results of a phylogenetic analysis, which reveals that *S. marcescens* Bar86 gene *SmchiC* is clustered within the bacteria chitinase group, and it has a closer relationship with *S. marcescens*. Meanwhile, the result of the phylogenetic analysis suggested that all chitinases evolved from the same ancestor, and that *SmchiC* shared a common evolutionary origin with chitinases from other bacteria (Figure 1b).

### 2.2. Cloning of Chitinase Genes

The *SmchiC* gene was amplified from *S. marcescens* Bar86 genomic DNA using specific PCR primers based on the coding sequences of known *S. marcescens* ChiC proteins. PCR amplification resulted in a product of 1443 bp, correlating with the size of coding sequences of known *S. marcescens* chitinolytic proteins in the current GenBank database.

### 2.3. Tobacco Transformation and Molecular Analysis

**Figure 2:**
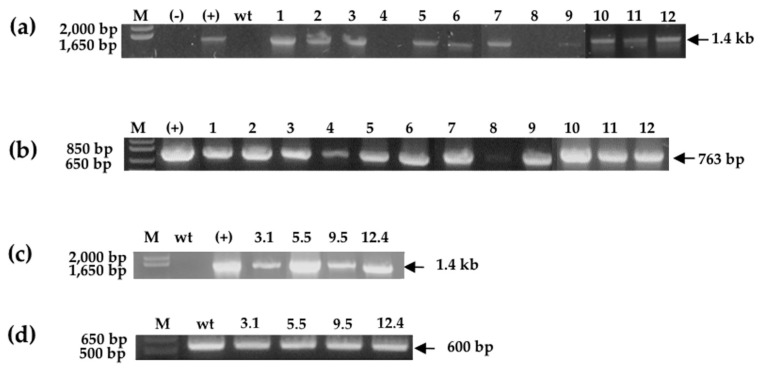
Molecular analysis of tobacco transgenic plants. (**a**,**b**) Primary transformant plants were analyzed for the presence of *SmchiC* and *nptII* genes by PCR amplification. Lane 1–12; transgenic tobacco lines, (-) water amplification control; (+) positive control (p2x35SChiC) vector; WT wild-type plant (negative control); M, molecular markers. (**c**,**d**) chitinase and actin expression by RT-PCR in homozygous transgenic plants. Transformed tobacco lines, namely lines 3.1, 5.5, 9.5 and 12.4; wt wild-type plant (negative control); M, molecular marker.

After transformation, which was mediated by the agrobacterium-method [30], from 36 kanamycin-resistant primary transformants 11 were positive for the amplification of *SmchiC* gene. All of them showed the amplification expected size of 1443 bp (Figure 2a) confirming transgenic events, and also the 763 bp PCR fragment of the selectable marker conferring resistance to kanamycin corresponding to *nptII* gene was corroborated (Figure 2b). Furthermore, RT-PCR analysis confirmed that the *SmchiC* gene was transcribed, and three transgenic lines were selected with low (3.1), medium (12.4) and high (5.5) expression levels, respectively (Figure 2c). The actin gene was used as housekeeping gene control (Figure 2d).

### 2.4. Western Blot Analysis 

**Figure 3:**
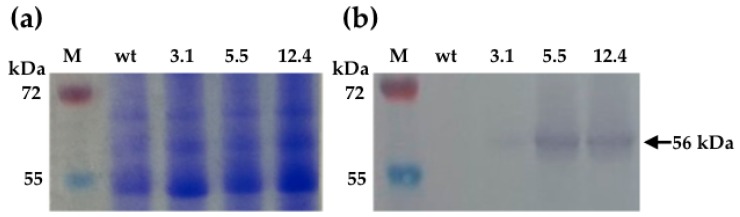
Detection of SmChiC protein in tobacco leaf extracts subjected to immunoblot analysis by Western blot. (**a**) SDS-PAGE analysis of crude leaf extracts, homozygous transgenic tobacco lines 3.1, 5.5 and 12.4; wt wild-type plant (negative control); M, protein molecular weight marker. (**b**) Western blot assay of crude leaf extracts revealing a band of 56 kDa corresponding to SmChiC endochitinase.

Detection of ChiC protein in tobacco was carried out by Western blot, using a polyclonal antibody against SmChiC. Immunoblots showed the presence of a prominent 56 kDa band corresponding to the size of the native bacterial chitinase, indicating that the transgene was expressed constitutively in the heterologous tobacco system. Endogenous chitinases were not detected by the antibody (wt, negative control). Lines of the homozygous progeny showed the highest protein expression (5.5 and 12.4 lines); line 3.1 was hint low (Figure 3a,b).

### 2.5. Endochitinase Activity

**Figure 4:**
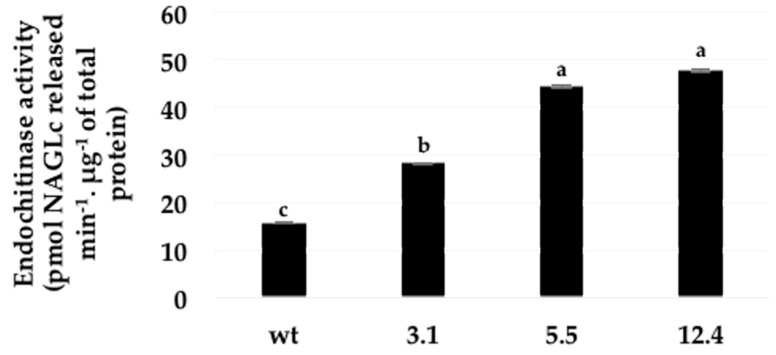
Endochitinase activity in crude leaves extracts of three different transgenic lines and untransformed (wt) plant. Error bars indicate standard deviation of means (SD). Within each frame different letters indicate statistically significant differences among transgenic lines (*p* = <0.0001), *n* = 4.

The chitinase activity in the leaves of transgenic and control plants was significantly higher in the three different transgenic tobacco lines (3.1, 5.5, and 12.4): but the lines 5.5 and 12.4 showed the highest activity (Figure 4).

### 2.6. Antifungal Inhibition Activity Assays on Crude Protein Extracts

**Figure 5:**
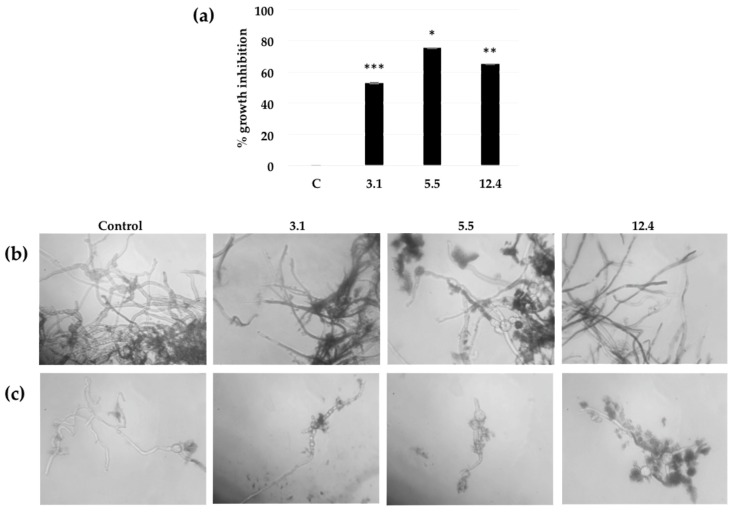
The effect of chitinase SmChiC protein in leaf extracts from homozygous transgenic tobacco plants over *B. cinerea* growth and spore germination. (**a**) After 48 h of incubation, percentage of fungal growth inhibition is defined as 100× the ratio of the corrected A_595_ of the control plant minus the corrected A_595_ of the sample over the corrected A_595_ of the control plant. Each bar value represents the mean ± SD of triplicate experiments (Student’s *t* test; ***, *p* = 0.0002; *, 0.0165; **, 0.0053 for each line respectively versus control). (**b**) Microscopic analyses of the appearance of the *B. cinerea* hyphae after incubation for 48 h in presence of different plant crude extract. Magnification target (40×). (**c**) Microscopic analyses of the appearance of *B. cinerea* single spore hyphae after incubation for 48 h in presence of different plant crude extract. Magnification 40×.

In vitro assays to quantitatively establish the fungal inhibition of the bacterial chitinase present in the leaf extracts of the transgenic lines were conducted on crude protein extracts from such three lines that showed expression of SmChiC at different levels (Figure 3b). The transgenic line 3.1 presented a moderately growth inhibition of 52.8 % against *B. cinerea*, whereas the other two lines 12.4 and 5.5 exhibited growth inhibitions of 65.1% and 75.4%, respectively against the same *B. cinerea* spores (Figure 5a).

The effect of the plant crude protein extracts on spore germination and hyphal development of *B. cinerea,* was microscopically observed after 48 h of incubation. Structural and physiological damage within the hyphae was observed when using the plant crude protein extracts of lines 12.4 and 5.5, which showed highest growth inhibition. The tobacco line 3.1 which had fairly low percentages of growth inhibition (52.8%), also showed less formation of fungal biomass compared to the effect of the leaf extract from control plants (non-transformed), where the germination and growth of hyphae is normal (Figure 5b,c). 

### 2.7. Botrytis Cinerea Resistance Assays on Detached Leaves

**Figure 6:**
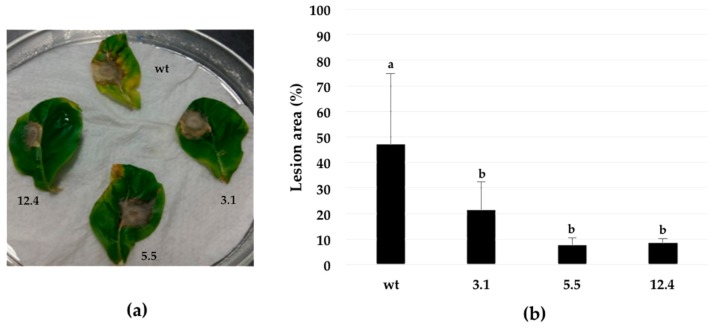
Resistance assay against phytopathogenic fungus *B. cinerea*. (**a**) Phenotype of transgenic plants after inoculation with mycelium of *B. cinerea* during 7 days. (**b**) Percentage of area leaf infection in control and transformed tobacco lines. Leaf damage by *B. cinerea* infection was significantly different from the control with a value at *p* < 0.0001. Different letters indicate statistically significant differences. Error bars indicate standard deviation of means (SD). Three independent replicates were performed for each experiment.

Resistance against the fungal pathogen *B. cinerea*, was evaluated in homozygous *N. tabacum* plants using a detached leaf test. An agar disc (0.2 cm diameter) containing mycelia of *B. cinerea*, was placed on the adaxial surface of leaves. Seven days after inoculation, symptoms on the three transgenic lines (3.1, 5.5, and 12.4) included mild chlorosis or a hypersensitive-like necrosis. In contrast, leaves from untransformed plants showed extensive areas of necrosis signs surrounded by chlorotic halos (Figure 6a). The lesion areas were reduced in the transgenic plants, when compared to the wild type line. Data indicate that transgenic plants are less susceptible to *B. cinerea* (Figure 6b).

### 2.8. Insect Toxicity Assay

**Figure 7:**
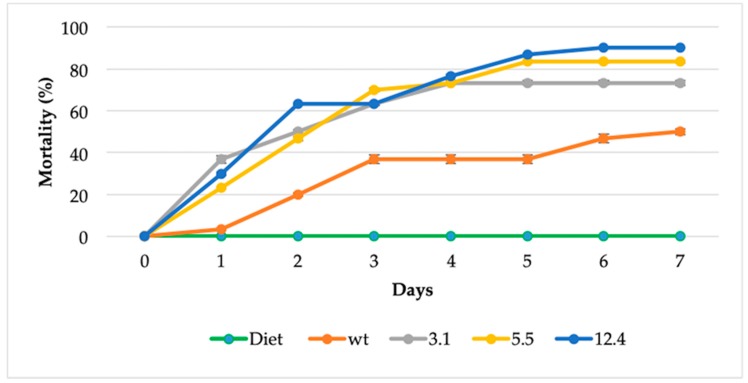
Toxicity of transgenic plants to *S. frugiperda* larvae on a plant-feeding assay. The data shown represent the mean of three independent experiments, and error bars indicate standard deviation of means (SD). Each experimental group was composed of 10 larvae and larvae mortality is reported as a percentage of the initial number of larvae. Diet is food for rearing *S. frugiperda*. The survival curve was significantly different from transgenic lines versus non-transformed plant (One way ANOVA, Tukey’s test, *p* < 0.0001).

Estimation of the toxicity of transgenic plants to insects was carried using a plant-feeding assay. Neonate larvae of fall armyworm *S. frugiperda* were fed in the laboratory with three different homozygous transgenic lines and non-transformed tobacco plant was used as a control (Figure 7). A significant difference on insect mortality were observed since the first day and until the end of the experiment (day 7), transgenic lines significantly increased mortality rate in neonate larvae [12.4 transgenic line = 90% (27 of 30 larvae died), 5.5 transgenic line 83% (25 of 30 larvae died), 3.1 transgenic lines 73% (22 of 30 died) vs. control 50% (15 of 30 larvae died)]. Most of the larvae that fed on transgenic leaves for 7 d died (Figure 7).

## 3. Discussion

Plant chitinases play an integral role in the innate plant resistance to pest and diseases [4]. These enzymes form part of the PR proteins in plants and have been extensively used in modern biotechnology [18,31,32] to evaluate their potential to increase plant’s resistance through genetic transformation technologies [13,33,34,35]. Chitinolytic bacteria of the genus *Serratia* are well known for their chitinase production [36,37]. Their ability to produce these hydrolytic enzymes could be considered an important virulence factor for contributing significantly as entomopathogen [26] as an effective biocontrol agent [38,39]. 

In this study, we have evaluated a bacterial endochitinase, which to the best of our knowledge has not been studied before in transgenic plants. Here, the *SmchiC* gene from *S. marcescens* was PCR-amplified from genomic DNA and cloned into a plant expression vector conferring constitutive expression in transformed tobacco plants. 

Eleven transgenic lines F_0_ were obtained with normal growth, which did not show differences compared with control tobacco plants (data not shown). This result is in agreement with earlier reports where expression of endochitinase did not adversely affect plants [7,13]. Plant selection continued until homozygous generation was reached and confirmed that they have integrated the transgene into their genome. RT-PCR analyses allowed the selection of low, medium and high transcript expression. These homozygous plants were used for further studies and was verified that the expression level is related to plant tolerance in different bioassays. The transcripts yielded active bacteria chitinase proteins that exhibited significant increases in endochitinase activities in the three different transgenic lines when compared with untransformed controls (Figure 4); these events have been already observed with chitinase-encoding genes by improving plant defense [13,40]. Here, transgenic lines showed less sensitivity to *B. cinerea* infection, as observed in transgenic cotton plants [41] and tobacco plants [7] expressing fungal chitinases. 

In vitro assays proved that the antifungal activity of the transgenic lines lead to growth inhibition (52–75%) in all three transgenic lines (Figure 5), similar results have been previously observed were tobacco plants transformed with a *S. cerevisiae* chitinase showed strong antifungal activity, leading to fungal growth inhibitions of 25–70% in various transgenic lines [42]. *In planta* assays demonstrated that leaves of transgenic lines infected with *B. cinerea* were capable of withstanding the fungal infection by decreasing disease susceptibility according to lesion measurements and chlorosis appearance, unlike untransformed control plants (Figure 6). According to our results, the improved tolerance against the fungal pathogen observed in this study is not a unique consequence of their chitinolytic activity. Chitin residues of cell wall or apoplastic glycoprotein-derived oligomers released by the action of chitinase act as the elicitor molecules and induce the plant defense mechanism, which include increased lignifications, phytoalexin synthesis, generation of reactive oxygen species and expression of PR-proteins [7,43]. Although the biosynthetic origin of these oligomers has not been well established, there is some evidence that they might derive from partial hydrolysis of apoplastic N-linked glycoproteins [44,45].

Finally, feeding assays were performed to evaluate the insecticidal potential of the transgenic plants expressing a bacterial chitinase towards neonate larvae of *S. frugiperda* and enhanced mortality was observed within the first day (Figure 7). Transgenic lines showed a significant difference on insect mortality of 90% by the seventh day. Other studies have shown that overexpressing a chitinase from *S. littoralis* showed a larvae mortality of 63–70% [28]. The entomopathogenic activity of *S. marcescens* (strain SEN) was comparable to that of *B. thuringiensis* treated larvae, which showed insecticidal activity against all the developmental stages of *S. litura* larvae [26]. 

These bioassays have demonstrated that transgenic plants overexpressing bacterial chitinase, have the capability to contend against fungal infection most likely due to its capacity to degrade the linear polymer of chitin (Figure 4) consisting of β-1, 4 N-acetylglucosamine and in fungi, by affecting structural parts like mycelia (Figure 6) and in case of insects over integral parts like cuticle and peritrophic matrix (PM). In the latter, probably by cleaving chitin present in the peritrophic membrane of the insect gut causing perforations, leading to disease and subsequent death of the infected larvae (Figure 7) [46]. Hence transgenic tobacco plants carrying the endochitinase *SmchiC* gene in their genome were protected from pathogenic fungi *B. cinerea* and from the pest insect *S. frugiperda.*

## 4. Materials and Methods

### 4.1. Materials

A *S. marcescens* isolate was kindly provided by Dr. Guadalupe Peña-Chora, Centro de Investigaciones Biológicas (CIB), Universidad Autónoma del Estado de Morelos (UAEM). Bacteria were isolated from a corpse of a larvae of the sugar cane borer *Diatraea magnifactella* (Lepidoptera: Pieridae) collected in the field.

### 4.2. Screening of Bacterial Isolate for Chitinolytic Aactivity

Chitinolytic activity of the isolated bacteria was determined using nutrient agar medium supplemented with colloidal chitin (0.05%) [47]. Luria Broth (LB; Q-Biogene, Carlsbad, CA, USA) was used for cultivation of the strain. *S. marcescens* culture was incubated by shaking (200 rpm) at 30 °C overnight and 4 µL of the culture was dropped onto a LB agar plate and after five days of incubation at 30 °C, colonies showed halo zones and maintained on nutrient agar plates. 

### 4.3. Bacterial Identification 

Genomic DNA was extracted from the selected bacterial isolate Bar86 using PUREGENE^®^ DNA Purification Kit. For bacterial identification, 16S rDNA was amplified using the universal bacterial primers 63F (5′-CAG GCC TAA CAC ATG CAA GTC-3′) [48] and L1401 (5′-CGG TGT GTA CAA GAC CC-3′) [49]. Amplification was performed under the following conditions: one cycle at 95 °C, 5 min; 37 cycles of 95 °C, 50 s; 59 °C, 50 s; 72 °C, 1 min; and one cycle at 72 °C, 10 min. PCR products were purified and sequenced at the DNA core facility of the Institute of Biotechnology, UNAM, Cuernavaca, Morelos, Mexico. 

The nucleotide sequences were aligned using CLUSTALW, and phylogenetic inferences for 16S rDNA and chitinases from other organisms, including plants, bacteria, fungi, and insects obtained using the maximum-likelihood method within the MEGA6 software with the neighbor-joining method. The sequence of the 16S rDNA gene was deposited in the GenBank with the accession number MH644845.

### 4.4. Chitinase Gene Isolation 

*SmchiC* gene coding for the 55-kDa endochitinase was amplified by PCR using the primer pair ChiC-F (5′ GGATCC ATG AGC ACA AAT AAC ACT AT 3′, *BamHI* site underlined) and ChiC-R (5′ GAGCTC TTA GGC GAT GAG CTG CCA CAG 3′, *SacI* site underlined), designed based on consensus sequences derived from *S. marcescens* chitinase *chiC* nucleotide sequences available in GenBank. Amplification was performed under the following conditions: one cycle at 95°C, 5 min; 28 cycles of 95 °C/45 s; 58 °C/45 s; 72 °C/60 s and one cycle at 72 °C/3 min. PCR product was cloned into pTZ57R/T cloning plasmid, thus generating the intermediate vector pTZchiC. The orientation within the plasmid and open reading frame integrity of the bacterial chitinase gene were confirmed by DNA sequencing, by means of automatic DNA sequencing using both M13 primers. The sequence was deposited in the GenBank with the accession number MH646662.

### 4.5. Tobacco Transformations

Transformation vector p2x35SchiC was constructed in the following manner. The *BamHI-SacI* fragment (1.6 Kb) from pTZchiC containing *chiC* gene was excised and cloned into the same sites of p2x35SNOS, between the double CaMV 35S promoter to obtain the plant transformant construct named p2x35SchiC. This vector was mobilized into the *Agrobacterium tumefaciens* strain LBA4404 by the electroporation method. 

*Nicotiana tabacum* cv. Petit Havana was used for the experiments. Transformation of *N. tabacum* was done according to the previously reported method [30]. Disc leaf explants of 7 mm size from in vitro grown shoot cultures of *N. tabacum* was used for transformation using *Agrobacterium tumefaciens* LBA4404 harboring the recombined plasmid p2x35SchiC. After co-culture, explants were transferred to Murashige and Skoog’s [50] basal medium supplemented with kanamycin (100 mg L^−1^) for selection of transgenic shoots and 500 mg L^−1^ of cefotaxime to eliminate the *Agrobacterium*. Putative Kanamycin-resistant transformants with well-developed roots were transferred to pots containing vermiculite and grown under greenhouse conditions. The presence and expression of the transgenes in primary transformants (T0), was verified by PCR analysis. Transgenic T3 homozygous plants harboring single copy integrations of transgenes were used for biotic functional analysis.

### 4.6. Molecular Analyses of Tobacco Transgenic Lines

To confirm, the transgene presence and integration DNA isolation from putative transgenic plants was achieved using the hexadecyltrimethyl ammonium bromide (CTAB) protocol. Leaf material (100 mg) was ground to a fine paste with 500 µL of CTAB buffer (Tris 1 M pH 8.0, EDTA 0.5 M pH 8.0, NaCl 5 M, PVP 40) using a pestle. Plant extract mixture was incubated for 15 min at 55 °C and after incubation, the sample was centrifuged at 12,000× *g* for 5 min to spin down cell debris. To the supernatant was added 250 μL of chloroform:isoamyl alcohol (24:1), followed my mixing by inversion and centrifugation at 13,000 rpm for 1 min. The aqueous phase was transferred into a new centrifuge tube (contains the DNA) and diluted with 50 μL of 7.5 M ammonium acetate followed by the addition of 500 μL of ice-cold absolute ethanol. The mixture was centrifuged (13,000 rpm for 1 min) and the pellet was resuspended in 100 µL of DNase-free water.

To confirm the transgene presence and integration PCR amplification analysis for the *NPTII* gene was conducted using the primers nptII_F (5′-GAACAAGATGGATTGCACGC-3′) and nptII_R (5′-GAAGAACTCGTCAAGAAGGC-3′) with an expected band size of 763 bp (35 cycles; 35 s at 94 °C; 5 min 94 °C; 45 s 57 °C and the primers used to amplify the SmchiC_F (5′GGATCC ATG AGC ACA AAT AAC ACT AT 3′) and chiC_R (5′GAGCTC TTA GGC GAT GAG CTG CCA CAG 3′), with an expected band size of 1.4 kb (35 cycles; 3 min at 95 °C; 45 s 95 °C; 45 s 58 °C).

Transgenic lines harboring the transgene were assayed by RT-PCR to qualitatively see the expression at mRNA level. A total RNA extraction was performed with TRIZOL reagent (Invitrogen, Carlsbad, CA, USA) according to manufacturer’s protocol.

### 4.7. Progeny Segregation

The primary transgenic lines were grown and self-pollinated in a greenhouse. T2 seeds of each line were surface sterilized and placed on Petri plates containing MS salts media (half-strength), supplemented with kanamycin (100 mg L^−1^). Seeds were germinated at 24 °C under a 16 × 8 h photoperiod for 14 d in a growth chamber. Seedlings were scored for their resistance to kanamycin and segregation was analyzed. Several T2 lines with single gene segregation for kanamycin resistance were grown in a greenhouse for flowering and their seeds were analyzed for segregation of the marker. Those lines that showed 100% resistance to kanamycin were considered homozygous. 

### 4.8. Preparation of Crude Protein Extracts from Transgenic Tobacco Lines

To extract proteins, 3 g of leaf tissue were ground with extraction buffer which consisted of Trizma base 50 mM, magnesium acetate 10 mM, glycerol 10%, ethylenediaminetetraacetic acid (EDTA) 1 mM, and phenylmethylsulfonyl fluoride (PMSF) 1 mM. The samples were centrifuged at 7500 rpm −4 °C and the supernatant was precipitated with ice cold acetone. The samples were centrifuged to remove solvent and total proteins, pellets were dissolved in 25 mM sodium citrate (pH 6.0) buffer for the antifungal activity assay, or PBS buffer for the endochitinase activity assay and western blot assay. 

### 4.9. Western Blotting

Total protein extract (40 µg) was electrophoresed on 12% polyacrylamide gel and transferred to cellulose membranes, using a trans-blot wet transfer cell (Bio-Rad, Hercules, CA, USA) overnight at 30 V. After protein blotting, the membrane was blocked by soaking in milk–based blocking buffer (5% powdered milk in 0.5% Tween 20) for 2 h at room temperature and then probed overnight at 4 °C temperature with the anti SmChiC antibody (1:2000 dilution in blocking buffer) as a primary antiserum. The membrane washed three times with TBST to remove the antibody excess, and then the immune-reactive protein bands were visualized using alkaline phosphatase–conjugated anti-rabbit (IgG-H+L) as a secondary antibody (5:10,000 dilution in blocking buffer) for 1 h. Three final TBST washes to remove excess antibodies were done and then equilibrated with APB buffer for 15 min. The alkaline phosphatase activity was determined by incubating the membrane at room temperature into an equal volume of 5-bromo-4-chloro-3-indolyl phosphate (BCIP) and nitro blue tetrazolium (NBT). 

### 4.10. Endochitinase Assay 

The dinitrosalicylic acid (DNSA) method was used to determine the chitinolytic activity [51] by measuring the concentration of N-acetylglucosamine released [52]. Reaction mixtures contained 0.5 mL of 1.5% colloidal chitin (in 10 mM KH_2_PO_4_ buffer, pH 7.0) and 0.5 mL of enzyme sample. The mixture was incubated at 50 °C for 1 h. The reaction was stopped by placing the tubes in a boiling water bath for 5 min. After cooling, the reaction mixture was centrifuged at 3000 rpm for 5 min. Then, 0.5 mL of the supernatant and 0.5 mL DNSA reagent were mixed together and incubated in a boiling water bath for additional 10 min. The absorption of the test sample was measured at 540 nm in a UV spectrophotometer (6405 UV/Vis, Jenway, UK) along with substrate and enzyme blanks. All measurements were performed for triplicate in each sample. One unit of chitinolytic activity was defined as the amount of enzyme required to produce 1 μmol N-acetylglucosamine per minute at 50 °C. The protein concentration was determined by the Bradford method [53] using bovine serum albumin as standard. 

### 4.11. Botrytis cinerea Spore Harvest for Antifungal Inhibition Assays

*B. cinerea* (B0510) was cultured on potato dextrose agar (PDA) medium (23 °C; dark) until sporulation occurred. Spores were prepared by a method modified from [54] by flooding the Petri dishes with sterile water and 0.05% of Tween 20. Plates were scraped to detach the conidia and the suspensions filtered through a funnel filled with cotton. Spore number was counted with a haemocytometer and adjusted to 1 × 10^5^ spores/mL and incubated on potato dextrose media (PD) for one hour.

### 4.12. Assays of Antifungal Inhibition Activity on Crude Protein Extracts

The inhibition assay was adapted from a previously reported method [55] and used to quantitatively determine the antifungal effect of the crude proteins in the leaf extracts from the transgenic lines on *B. cinerea* spore germination and fungal growth. The assays were performed in microtubes in a final volume of 1 mL. The crude protein samples 5 mg/mL constituted of 500 µL and 500 µL of potato dextrose broth (Difco, Franklin Lakes, NJ, USA) and *Botrytis* spores to a concentration of 4 × 10^4^ spores per mL. As a background control, 500 µL of 25 mM sodium citrate (pH 5.0) buffer was added to 500 µL of spore-containing PDB. The plates were incubated at 25 °C for 48 h. The absorbance at 595 nm (A) of the samples was determined every 24 h with a Jenway Spectrophotometer (6405 UV/Vis), starting at 0 h. Time zero values were used to normalize the 24 and 48 h values. These values are referred to as corrected A_595_ values. Growth inhibition is defined as 100× the ratio of the corrected absorbance (A_595_) of the control plant minus the corrected A_595_ of the sample over the corrected A_595_ of the control plant. To determine the condition of the *B. cinerea* hyphae, after 48 h of incubation, 10 µL of each sample was microscopically analyzed. 

### 4.13. Resistance Assays of Botrytis cinerea on Detached Leaves

For pathogenicity tests, cultures of *B. cinerea* (B0510) were cultured on PDA medium (23 °C; dark). When the fungal mycelia reached the edge of the plate, a 0.5 cm diameter agar plug with mycelium was removed from a region close to the edge using a cork borer and used for inoculations. Healthy and young leaves were collected of 4-week-old tobacco plants and placed on wet paper towels in Petri dishes. One agar plug was placed on each leaf containing *B. cinerea* mycelium were placed directly on the adaxial side of the leaves and incubated in the dark. After one-week leaves were photographed and the percentage of leaf area with necrosis was determined using ImageJ. 

### 4.14. Bioassays for Toxicity to Insects

Resistance assays against lepidoptera *S. frugiperda* was performed with *SmChiC*-overexpressing T3 plants using detached leaf method. In this method, detached leaves from control and transgenic lines were randomly selected and placed on moist paper into petri-plate. Neonate larvae were individually laid onto detached leaves of each transgenic and control plants for 7 d at at 25 °C under 12-12 light/dark regime. Bioassays were repeated three times using 10 larvae for each treatment. Mortality was scored daily. 

### 4.15. Analysis

The results are expressed as mean ± standard deviation. Statistical analyses were performed by one-way analysis of variance test with test by Tukey analysis or Student’s *t* test when appropriate. 

## 5. Conclusions

In the present study we have demonstrated that the endochitinase SmChiC protein normally found in *S. marcescens* Bar86 and overexpressed in transgenic plants, enhanced the tolerance against *B. cinerea* infection and can play a crucial role in defense against herbivorous insects such as *S. frugiperda* as an oral insecticide. The events presumably occur because of the augmented chitinase activities presented in tobacco plants, which hydrolyzes β-1,4 bonds of the structural chitin present in fungi and herbivorous insects. 

## Abbreviations

PR-	Pathogenesis-related
GlcNAc	β-1,4-*N*-acetylglucosamine
*S. marcescens*	*Serratia marcescens*
*B. cinerea*	*Botrytis cinerea*
*S. frugiperda*	*Spodoptera frugiperda*
*B. thuringiensis*	*Bacillus thuringiensis*
PM	Peritrophic matrix
